# KUS121, an ATP regulator, mitigates chorioretinal pathologies in animal models of age-related macular degeneration

**DOI:** 10.1016/j.heliyon.2018.e00624

**Published:** 2018-05-14

**Authors:** Yuki Muraoka, Yuto Iida, Hanako O. Ikeda, Sachiko Iwai, Masayuki Hata, Takeshi Iwata, Mao Nakayama, Nobuhiro Shimozawa, Yuko Katakai, Akira Kakizuka, Nagahisa Yoshimura, Akitaka Tsujikawa

**Affiliations:** a Department of Ophthalmology and Visual Sciences, Kyoto University, Graduate School of Medicine, Kyoto, Japan; b Department of Experimental Therapeutics, Institute for Advancement of Clinical and Translational Science, Kyoto University Hospital, Kyoto, Japan; c Division of Molecular and Cellular Biology, National Institute of Sensory Organs, National Hospital Organization Tokyo Medical Center, Tokyo, Japan; d Tsukuba Primate Research Center, National Institutes of Biomedical Innovation, Health and Nutrition, Tsukuba, Japan; e The Corporation for Production and Research of Laboratory Primates, Ibaraki, Japan; f Laboratory of Functional Biology, Kyoto University Graduate School of Biostudies & Solution Oriented Research for Science and Technology, Kyoto, Japan; g Department of Ophthalmology, Kitano Hospital, Osaka, Japan

**Keywords:** Cell biology, Neuroscience, Ophthalmology

## Abstract

Age-related macular degeneration (AMD) is a leading cause of blindness among elderly people. The appearance of drusen is a clinical manifestation and a harbinger of both exudative and atrophic AMD. Recently, antibody-based medicines have been used to treat the exudative type. However, they do not restore good vision in patients. Moreover, no effective treatment is available for atrophic AMD. We have created small chemicals (Kyoto University Substances; KUSs) that act as ATP regulators inside cells. In the present study, we examined the *in vivo* efficacy of KUS121 in C-C chemokine receptor type 2-deficient mice, a mouse model of AMD. Systemic administration of KUS121 prevented or reduced drusen-like lesions and endoplasmic reticulum stress, and then substantially mitigated chorioretinal pathologies with significant preservation of visual function. Additionally, we confirmed that long-term oral administration of KUS121 caused no systemic complications in drusen-affected monkeys. ATP regulation by KUSs may represent a novel strategy in the treatment of drusen and prevention of disease progression in AMD.

## 1 Introduction

Age-related macular degeneration (AMD) is an eye disease whose main clinical manifestation is the gradual and progressive loss of central vision. AMD is a leading cause of blindness in elderly people worldwide, and its prevalence is still increasing, especially in developed countries [1]. AMD is a multifactorial disease; the main risk factor is aging [2], although hereditary predisposition and environmental factors also affect the pathogenesis and progression of the disease [3]. In terms of the underlying disease mechanism, it has been proposed that endoplasmic reticulum (ER) stress causes functional loss in the retinal pigment epithelium (RPE) [4, 5] and subsequent neuronal cell death in the retina.

Late-stage AMD (late AMD) is classified into two types, exudative and atrophic AMD, based on pathology [1]. The exudative type is characterized by the presence of newly created, fragile choroidal neovascular vessels. Thus, it is often accompanied by retinal edema, subretinal fluid accumulation, or even hemorrhage in the advanced stages. These symptoms directly or indirectly impair the function of the macula, namely central vision. Atrophic AMD is characterized by atrophic degeneration—known as geographic atrophy—across a wide area of the retina. Regardless of the type of late AMD, characteristic deposits known as drusen are often observed in the early or even preclinical stages of AMD [6]; these are extracellular accumulations of lipoproteins between the RPE and Bruch's membrane. In addition, “drusenoid deposits” [7]—another type of characteristic subretinal accumulation—are observed in 50% of patients with early and intermediate AMD [8]. Notably, about 55% of patients with subretinal drusenoid deposits are expected to develop late AMD of either the exudative or atrophic type [9].

To treat the exudative type of late AMD, antibody-based medicines (*e.g.* anti-vascular endothelial growth factor (VEGF) antibodies) have been used [10]. These drugs have led to transient improvements in exudative lesions from the neovascular vessels. However, once the foveal photoreceptors and RPE have been damaged by neovascularization, the visual prognosis is generally poor, even if the therapy is aggressively applied. On the other hand, no treatment is currently available for atrophic AMD [11]. To achieve a better visual prognosis or prevent progression to late AMD outright, a new strategy targeting the earlier stages of the disease, such as an amelioration of RPE functions or a drusen-clearing therapy, has long been awaited.

Valosin-containing protein (VCP) is a ubiquitously expressed ATPase that is involved in neurodegeneration as well as physiological activities [12]. Recently, we have developed novel chemical compounds that specifically inhibit VCP's ATPase activity without affecting its cellular functions. We have referred to these chemicals as Kyoto University Substances (KUSs) [13]. KUSs can maintain cellular ATP levels and thus function as ATP regulators, and consequently suppress endoplasmic reticulum (ER) stress and cell death under various stressful conditions—both *in vitro* and in several pathological conditions *in vivo*. In fact, KUSs have been shown to mitigate retinal pathology in animal models of retinitis pigmentosa [13, 14], glaucoma [15], and retinal ischemia [16].

Because KUSs suppress ATP decreases and cell death in retinal cells, we decided to test whether these compounds can suppress pathology involving the RPE in animal models of AMD. Although no animal model reproduces all of the AMD-associated traits, C-C chemokine receptor type 2 (CCR2)-deficient mice have been reported to mimic the chorioretinal pathology of early and late AMD [17, 18]. In the present study, we administered KUS121, one of the KUSs, to CCR2-deficient mice and cynomolgus monkeys with drusen. We then investigated whether the compound affected the animals' pathologies.

## 2 Results

### 2.1 KUS121 prevents increases of round yellowish lesions in the fundus of CCR2-deficient mice

**Fig. 1:**
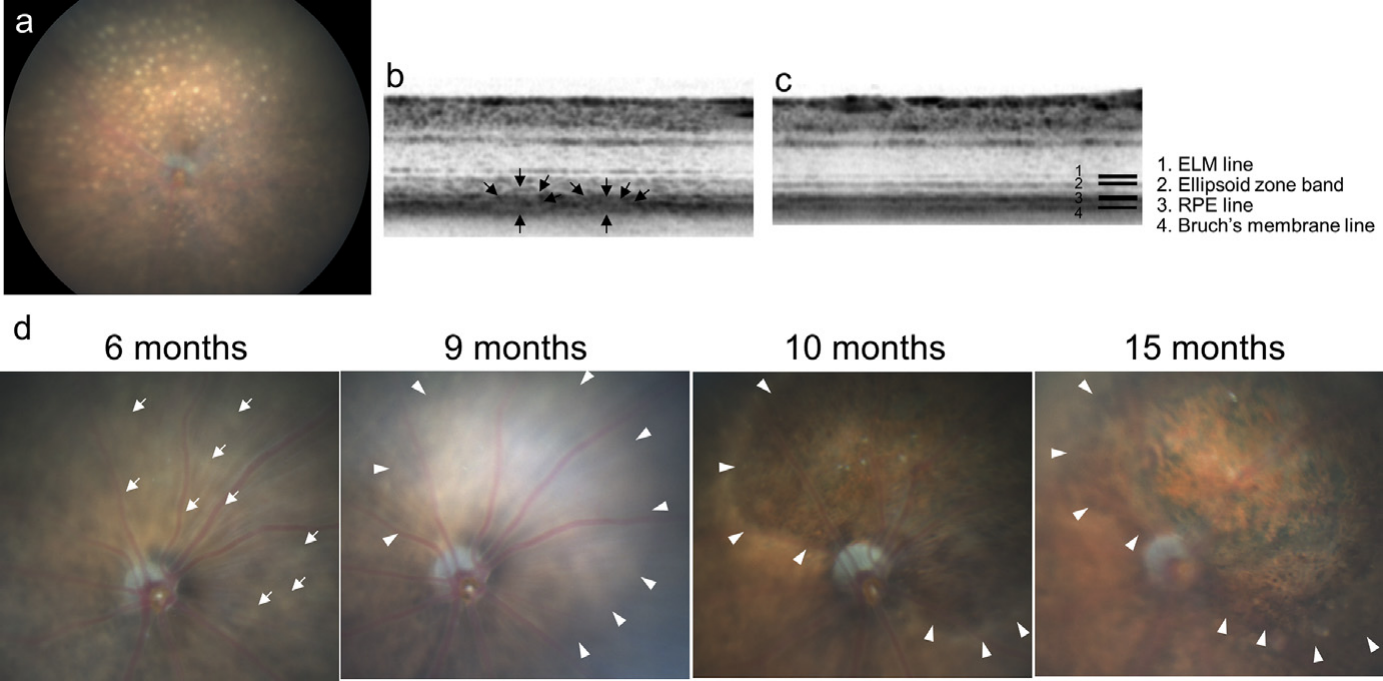
Characteristic features of fundus lesions in CCR2-deficient mice. a) A color fundus photograph of a CCR2-deficient mouse at age 9 months, showing numerous round yellowish lesions, each of which is approximately 1/4 disc diameter in size, and which are most clearly seen when focusing at the retinal pigment epithelium (RPE) level. b, c) Spectral-domain optical coherence tomography (SD-OCT) images of a 9-month-old CCR2-deficient mouse (b), and a wild-type mouse (c). The SD-OCT of the CCR2-deficient mouse shows moderate hyperreflectivity at the outer aspect of the photoreceptor layer and bulging in the photoreceptor and the RPE layers (arrows in b); this is consistent with the round yellowish lesions in color fundus photography. In contrast, the SD-OCT of the wild-type mouse shows four distinct lines at the chorioretinal interface (c). d) Time-dependent changes in color fundus photographs of a CCR2-deficient mouse at ages of 6, 9, 10, and 15 months. As the round yellowish lesions (arrows) expand and merge (arrowheads), retinal atrophy is evident at the corresponding retinal area.

Color fundus photography in aged CCR2-deficient mice [17] often showed round yellowish lesions that were each approximately 1/4 disc diameters in size and were most clearly seen at the RPE level (Fig. 1a). Sectional spectral-domain optical coherence tomography (SD-OCT) images always showed moderate hyperreflectivity at the outer boarder of the photoreceptor layer (Fig. 1b). This hyperreflectivity often made it difficult to precisely differentiate which retinal sites corresponded to the round yellowish lesions seen in color fundus photography. Nevertheless, SD-OCT did occasionally indicate bulging in the photoreceptor and the RPE layers that probably corresponded to the round yellowish lesions (Fig. 1b). Furthermore, longitudinal observation using color fundus photography showed that the round yellowish lesions expanded and sometimes merged as the mice aged, and that such developments were followed by degenerative changes (Fig. 1d).

**Fig. 2:**
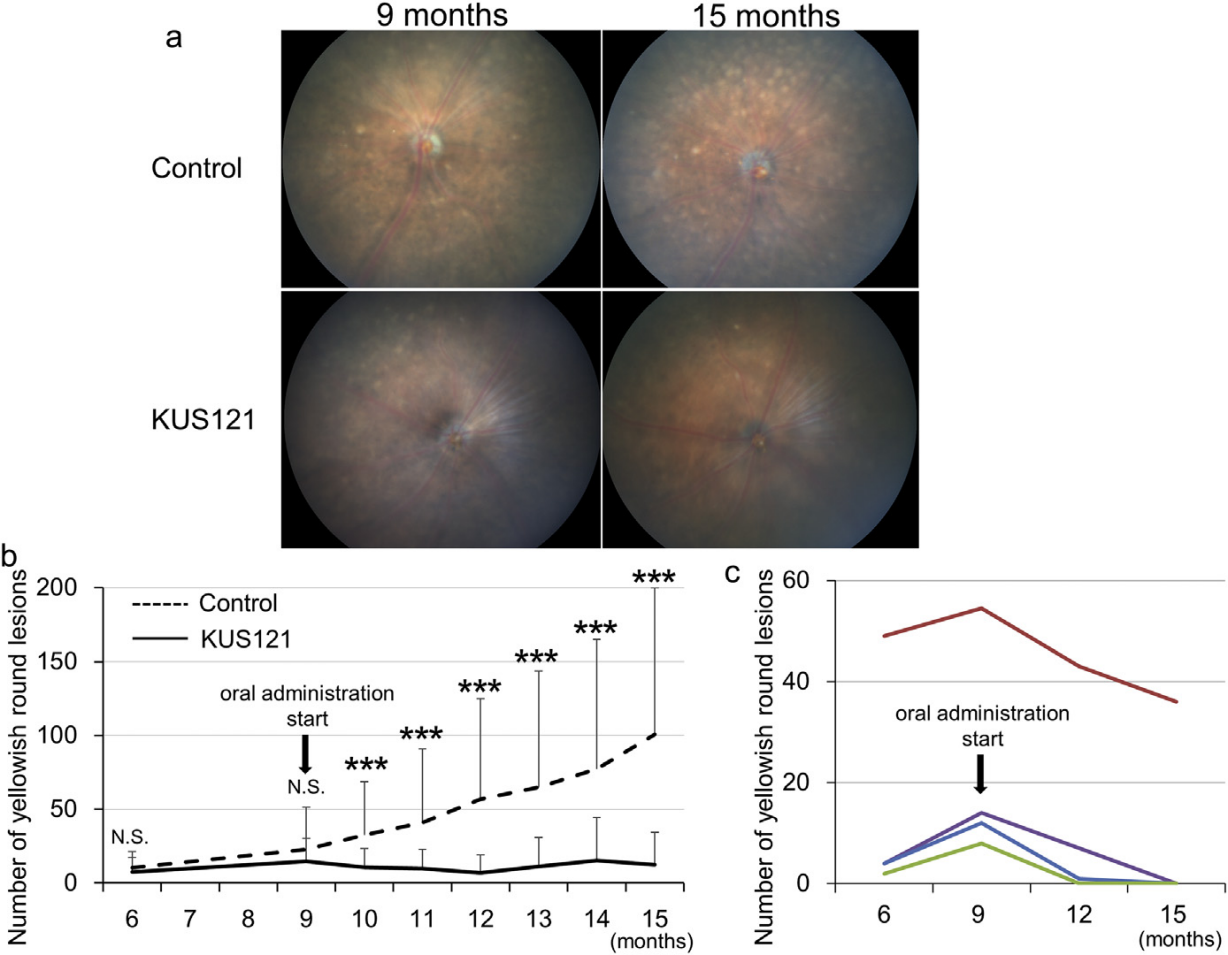
Effect of KUS121 on round yellowish lesions in CCR2-deficient mice. a) Representative color fundus photographs of CCR2-deficient mice with or without KUS121 treatment at the ages of 9 (start of administration) and 15 months (end of treatment). b) Comparison of longitudinal changes in round yellowish lesion numbers between CCR2-deficient mice with or without KUS121 treatment. N.S.: not significant, ***p < 0.005 vs. control (unpaired *t*-test). c) Longitudinal changes in round yellowish lesion numbers in four KUS121-treated CCR2-deficient mice.

Next, we examined the effects of KUS121 on the round yellowish lesions. We divided CCR2-deficient mice into two groups: a control group that received no treatment and KUS121-treated groups (11 and 15 mice, respectively). At the ages of 6 months and 9 months (when treatment began), there was no significant difference in the number of round yellowish lesions between the groups (22.8 ± 28.5 and 14.8 ± 15.4 at 9 months, respectively; Fig. 2a and b). The round lesions in the control group increased with age, but those in the KUS121-treated group appeared to remain unchanged or to increase only marginally (100.7 ± 99.3 and 12.3 ± 22.1 at 15 months, respectively). There were significantly fewer round yellowish lesions in the KUS121-treated group than in the control group after the age of 10 months (Fig. 2b). In four mice in the KUS121-treated group, the round yellowish lesions apparently decreased in number during the experimental period; no degenerative change occurred in these mice (Fig. 2c).

### 2.2 KUS121 mitigates age-dependent chorioretinal pathology in CCR2-deficient mice

**Fig. 3:**
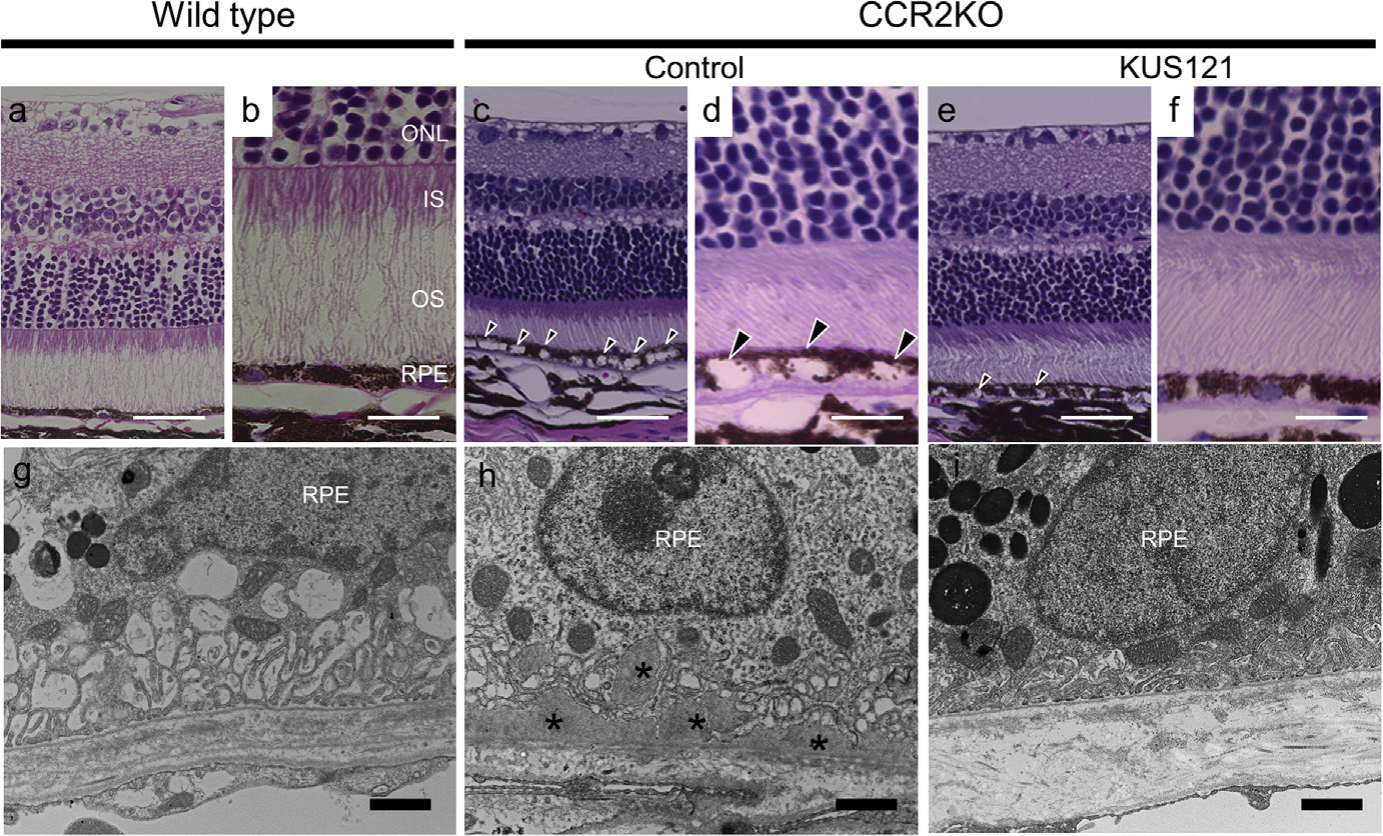
Effect of KUS121 on chorioretinal interface of aged CCR2-deficient mice. a–f) Hematoxylin and eosin-stained sections of wild-type mice (a and b) and age-matched 15-month-old CCR2-deficient mice (CCR2KO) without KUS121 treatment (control, c and d) or with KUS121 treatment (e and f, with treatment begun at 9 months of age). Arrowheads indicate vacuolization in the RPE cells. g–i) Transmission electron microscopy findings of 15-month-old wild-type mice (g), and age-matched CCR2-deficient mice (CCR2KO) with (i) or without KUS121 treatment (control, h). Asterisks (*) indicate basal laminar deposits in the interspaces of the RPE infoldings. IS, inner segment; ONL, outer nuclear layer; OS, outer segment; RPE, retinal pigment epithelium. Scale bars: 50 μm in (a), (c), and (e); 20 μm in (b), (d), and (f); 1 μm in (g), (h), and (i).

To examine the effect of KUS121 on the chorioretinal pathology of CCR2-deficient mice, we performed a morphological analysis on retinal sections from the mice. Hematoxylin and eosin-stained sections from 15-month-old wild-type mice showed well-organized microstructures in the photoreceptor and RPE layers (Fig. 3a and b). In contrast, sections from the control age-matched CCR2-deficient mice (control) showed widespread vacuolization in the RPE cells (Fig. 3c and d). However, in age-matched CCR2-deficient mice treated with KUS121, there was much less vacuolization in the RPE cells (Fig. 3e and f).

To examine the chorioretinal ultra-microstructure, transmission electron microscopy (TEM) sections were assessed. The TEM images of wild-type mice showed physiological infoldings without any deposits at the basal side of the RPE (Fig. 3g). In contrast, those of the CCR2-deficient control mice showed abundant basal laminar deposits within the interspaces of RPE infoldings (asterisks in Fig. 3h). These deposits appeared to correspond to the round yellowish lesions in color fundus photography (Fig. 2a). However, such basal laminar deposits were rarely observed at the basal side of the RPE in KUS121-treated CCR2-deficient mice (Fig. 3i).

Next, we measured the total retinal thickness in the SD-OCT images. The average retinal thicknesses of the control and the KUS121-treated mice were 257 ± 11 μm and 260 ± 14 μm at 13 months of age (n = 16 eyes and 26 eyes, respectively), which were not significantly different between the groups (*p* = 0.443, unpaired *t*-test).

### 2.3 KUS121 preserves visual function in CCR2-deficient mice

**Fig. 4:**
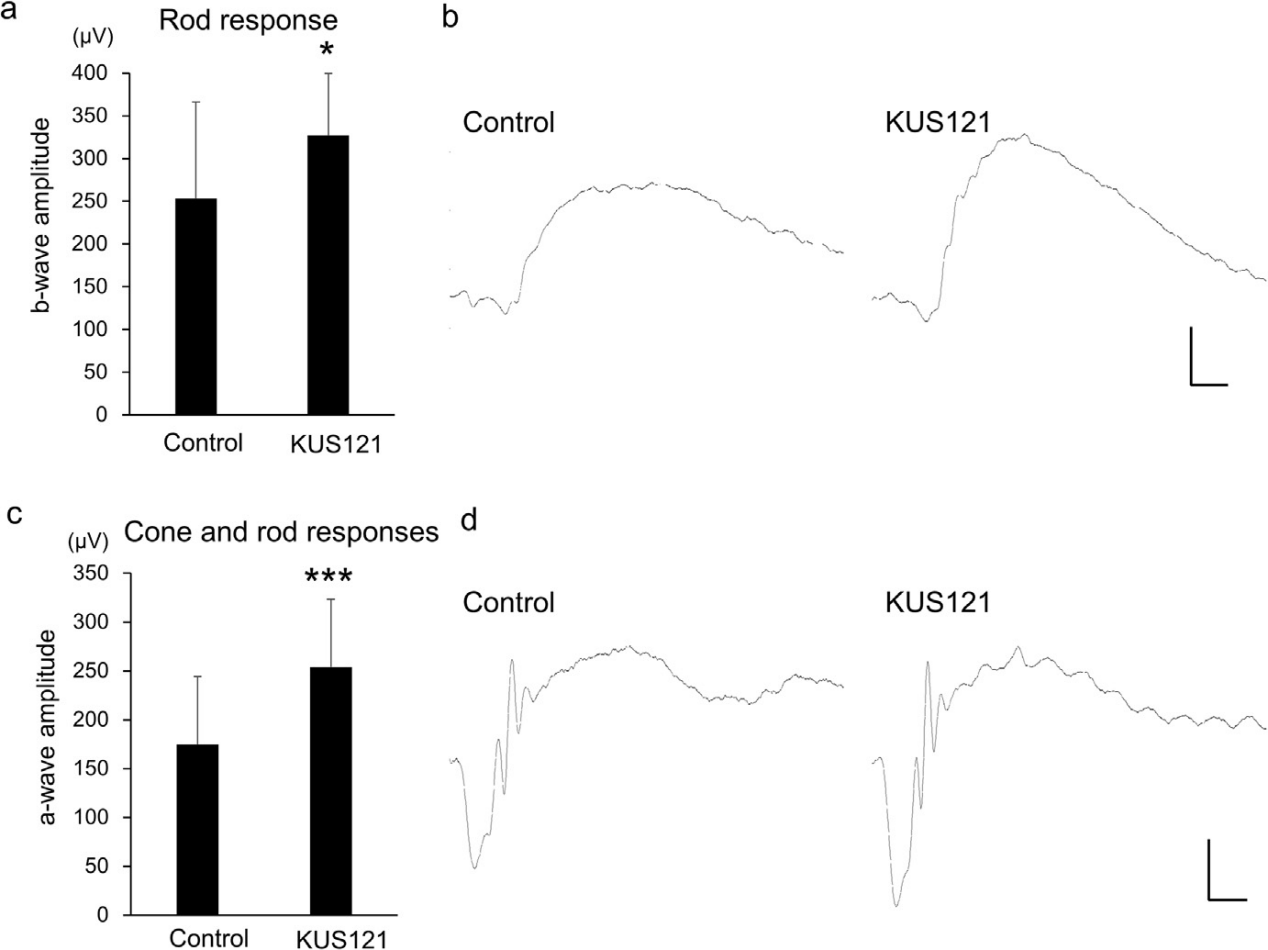
Effect of KUS121 on visual function in CCR2-deficient mice. Scotopic electroretinography in 9-month-old CCR2-deficient mice that had been treated with or without KUS121 for 7 months. a) b-wave amplitudes of the rod response (at a stimulus intensity of 0.01 cds/m^2^). b) Representative electroretinography from CCR2-deficient mice that produced the median b-wave amplitudes. c) a-wave amplitudes of the cone and rod responses (at a stimulus intensity of 3.0 cds/m^2^). d) Representative electroretinography from CCR2-deficient mice that produced the median a-wave amplitudes. *p = 0.028, ***p = 0.003 vs. control (unpaired *t*-test). Scale bar: 20 msec, 100 μV.

To investigate the effect of KUS121 on the retinal function of CCR2-deficient mice, we made another grouping of these mice and examined them using scotopic electroretinography (Fig. 4). In 9-month-old CCR2-deficient mice that had been treated with KUS121 for 7 months, the b-wave amplitudes of the rod response were significantly greater than those of non-treated CCR2-deficient mice (326.8 ± 72.6 μV vs. 253.1 ± 113.2 μV, respectively; *p* = 0.028 by unpaired *t*-test). Furthermore, the a-wave amplitudes of the cone and rod response in KUS121-treated CCR2-deficient mice were also significantly greater than those in non-treated mice (254.0 ± 69.6 μV vs. 174.6 ± 69.6 μV, respectively; *p* = 0.003 by unpaired *t*-test).

### 2.4 KUS121 prevents chronic ER stress in the retina and choroid of aged CCR2-deficient mice

**Fig. 5:**
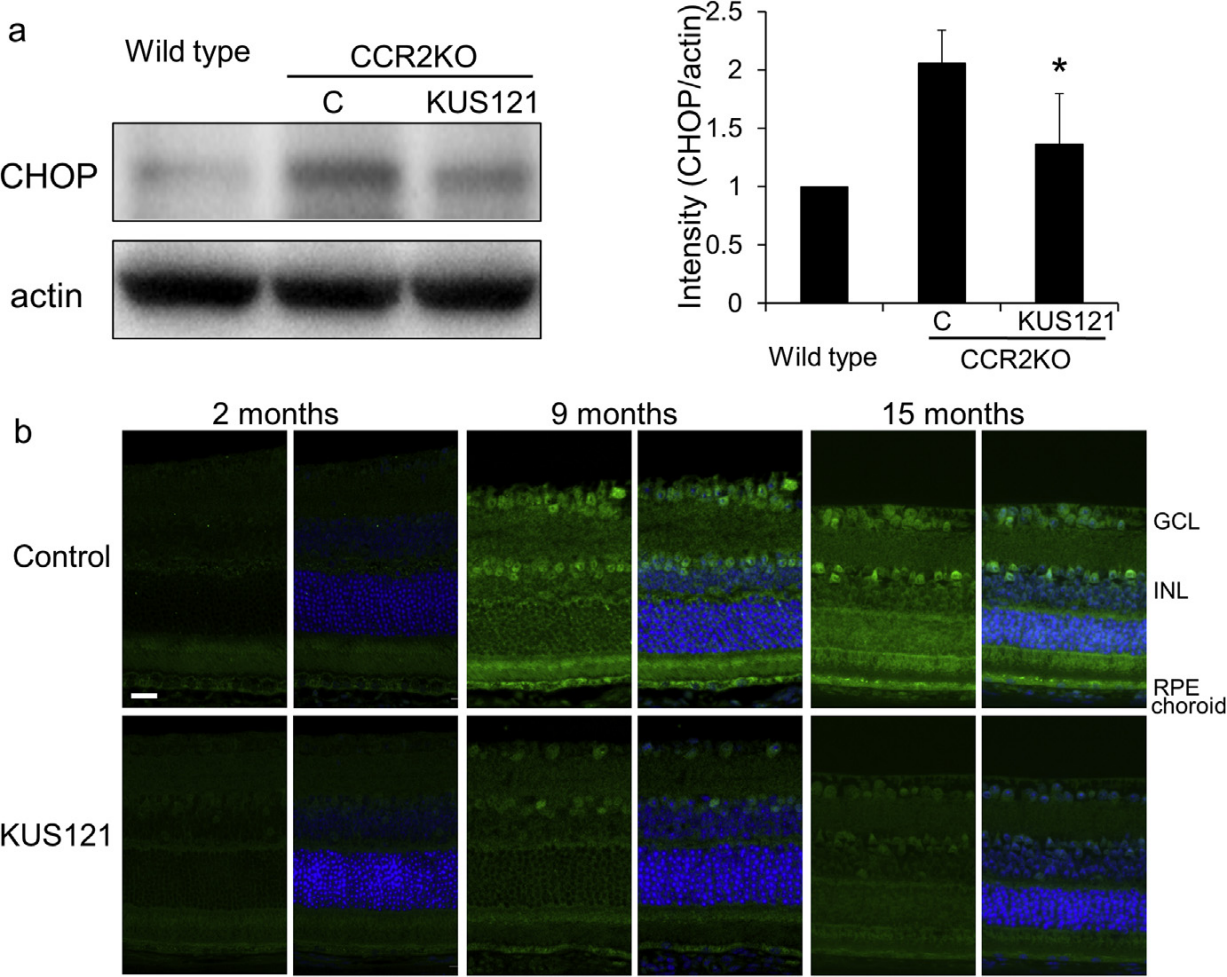
Effect of KUS121 on ER stress in chorioretinal tissues of CCR2-deficient mice. a) Western blot analysis of RPE and choroid mixed tissues from 15-month-old CCR2-deficient mice (CCR2KO) using a CHOP antibody with actin as a loading control. The ratios of CHOP to actin from four independent experiments are shown. Error bars indicate standard deviation. C: control. *p = 0.023 vs. control, Tukey's test. Complete scans of western blots are shown in Supplementary Fig. S1. Immunohistochemical study with a CHOP antibody (green) using chorioretinal tissues of albino CCR2-deficient mice without KUS121 treatment (control) and with KUS121 treatment at the age of 2 (start of administration), 9, and 15 months. Nuclei were counter-stained with DAPI (blue). GCL: Ganglion cell layer; INL: Inner nuclear layer; RPE: Retinal pigment epithelium. Scale bar: 20 μm.

We have reported that KUSs (e.g. KUS121 and KUS187) can suppress ER stress in the affected retina in mouse models of retinitis pigmentosa and glaucoma. Therefore, we next examined the effects of KUS121 on ER stress in CCR2-deficient mice. Specifically, we collected a tissue mixture of RPE and choroid from 15-month-old CCR2-deficient or wild-type mice and assessed ER stress using western blotting (Fig. 5a). The expression levels of C/EBP-homologous protein (CHOP), which is upregulated during ER stress [19], were significantly higher in the non-treated CCR2-deficient mice (control group) than in age-matched wild-type mice. In the KUS121-treated CCR2-deficient mice, the expression levels of CHOP were significantly lower than in the control group at the age of 15 months (*p* = 0.023 by Analysis of variance; Fig. 5a).

Immunohistochemical study of the chorioretinal tissues in non-treated CCR2-deficient albino mice (control group) showed age-dependent increases in CHOP-staining, not only in the choroid and RPE, but also in the inner retina (*e.g.* ganglion cell layer, and inner nuclear layer; Fig. 5b). In contrast, in age-matched KUS121-treated CCR2-deficient albino mice, in which treatment was started at 2 months of age, CHOP-staining was fainter, suggesting that KUS121 had strongly suppressed chronic age-induced ER-stress in the chorioretinal tissues of CCR2-deficient mice.

### 2.5 Administration of KUS121 to cynomolgus monkeys with drusen

**Fig. 6:**
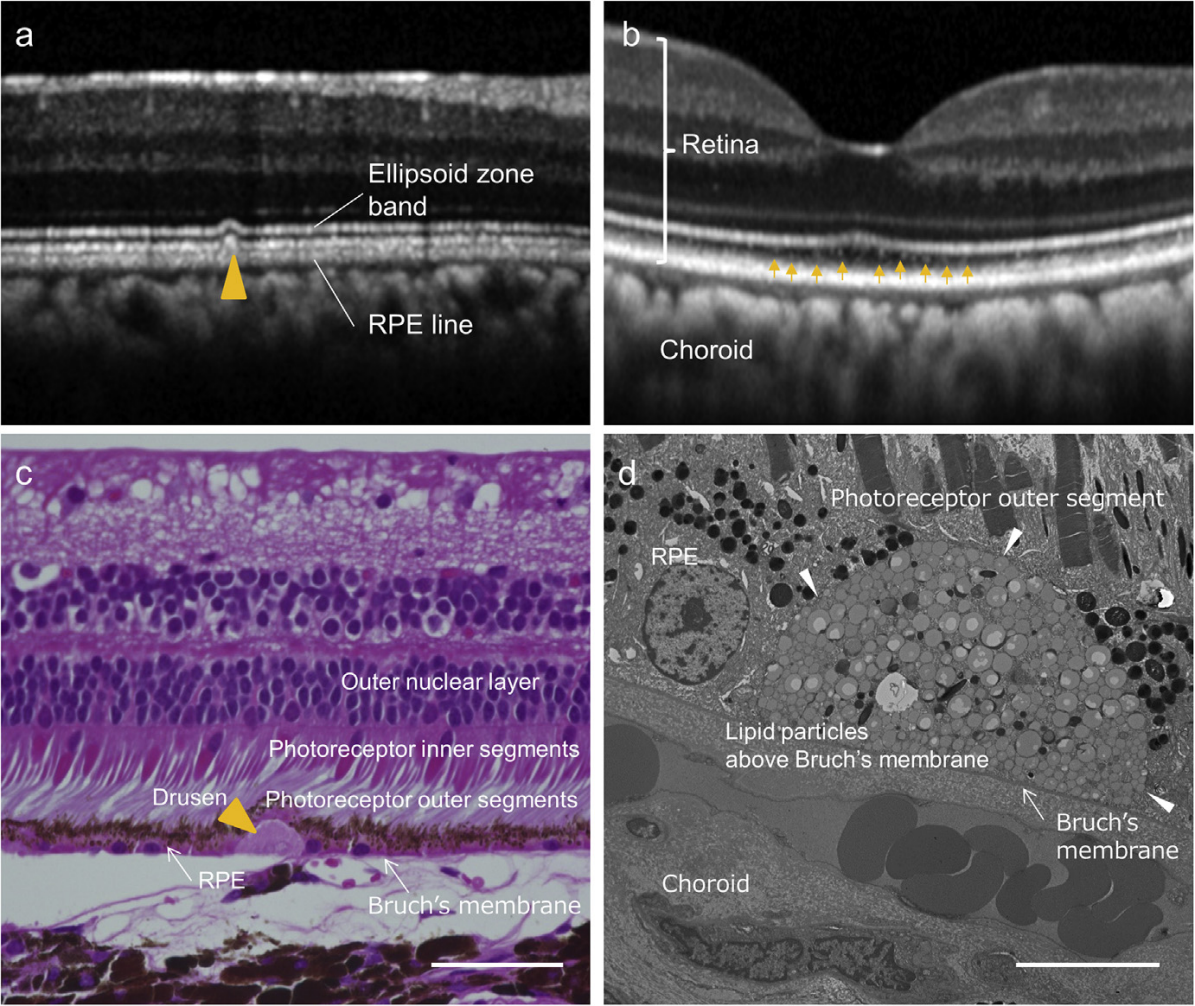
Drusen in cynomolgus monkeys. a,b) Spectral-domain optical coherence tomography (SD-OCT) sections of cynomolgus monkey retina. Drusen can be seen as moderate hyperreflective material (yellow arrowhead) accompanying bulging of the photoreceptor layer (a), as well as fine hyperreflective particles (arrows) between the ellipsoid zone band and the RPE layer at the fovea (b). c) A hematoxylin and eosin-stained section of retina and choroid of a cynomolgus monkey. Drusen can be observed as eosin-stained deposits between the RPE and Bruch's membrane (yellow arrowhead). d) A transmission electron microscopy section of a cynomolgus monkey eye. The ultra-microstructure within the drusen appears to be an aggregation of fine oil droplet-like materials (within white arrowheads). Scale bar: 50 μm in (c), 10 μm in (d).

As the animal model closest to human AMD, cynomolgus monkeys are widely used. Thus, we next performed experiments using five cynomolgus monkeys with drusen: 7, 8, 23, 23, and 26 year-old monkeys [20, 21]. A representative image of drusen on SD-OCT sections showed a moderate hyperreflective material accompanied by bulging in the RPE and photoreceptor layer (ellipsoid zone band, yellow arrowhead in Fig. 6a). Another image showed fine hyperreflective materials between the ellipsoid zone band and RPE layer at the fovea (arrows in Fig. 6b); these resembled the subretinal drusenoid deposits of AMD. Drusen in these cynomolgus monkeys was histologically observed as sub-RPE deposits above Bruch's membrane that stained uniformly with eosin (arrowhead in Fig. 6c). The ultra-microstructure within the drusen contained many aggregates of round oil droplet-like materials (Fig. 6d).

**Fig. 7:**
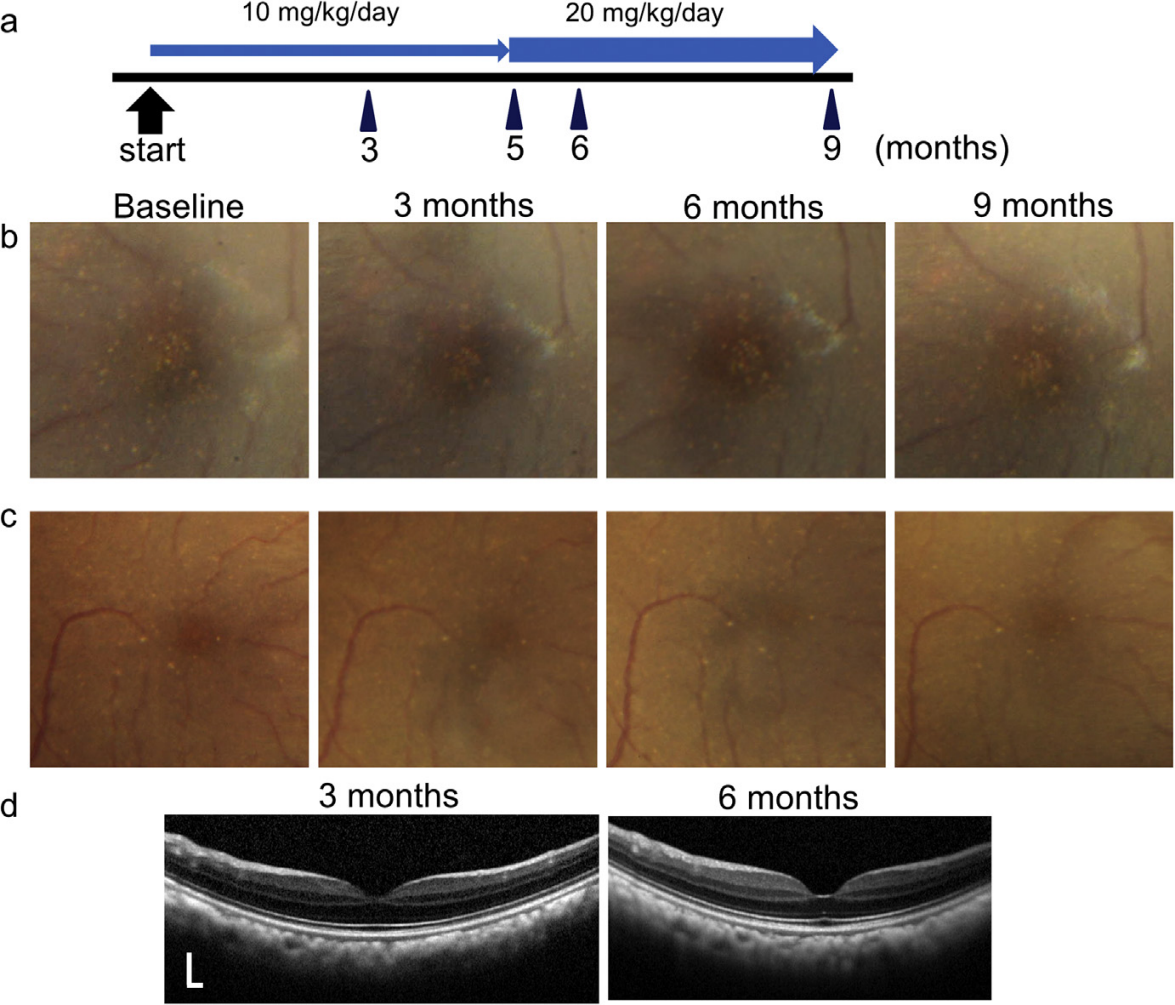
Longitudinal changes in drusen of cynomolgus monkeys. a) Schema showing time course of ophthalmic examination and dose of KUS121 administration. b) Color fundus photographs obtained from the left eye of an 8-year-old cynomolgus monkey with drusen at baseline and at 3, 6, and 9 months after the start of administration. c, d) Color fundus photographs (c) and SD-OCT images (d) obtained from the right eye of a 23-year-old cynomolgus monkey affected with drusen at baseline and at 3, 6, and 9 months (c) and at 3 and 6 months (d) after the start of administration. The drusen of the two monkeys seemed to be slightly decreased throughout the follow-up period. Scale bar: vertical and horizontal dimension, 200 μm.

The five monkeys were then administered KUS121 daily at a dose of 10 mg/kg/day (Fig. 7). Three months after the start of KUS121 administration, the number of macular drusen appeared to be unchanged from the baseline in three monkeys (7, 23, and 26 years old, respectively). However, in two monkeys (8 and 23 years old, respectively) drusen seemed to have slightly decreased (Fig. 7b and c). In these two monkeys, the dosage of KUS121 was then raised from 10 to 20 mg/kg/day from 5 months until the final evaluation point (9 months after the start of administration). The drusen of the two monkeys seemed to have decreased further, although only slightly, at the end point of the experiment (Fig. 7b and c).

**Fig. 8:**
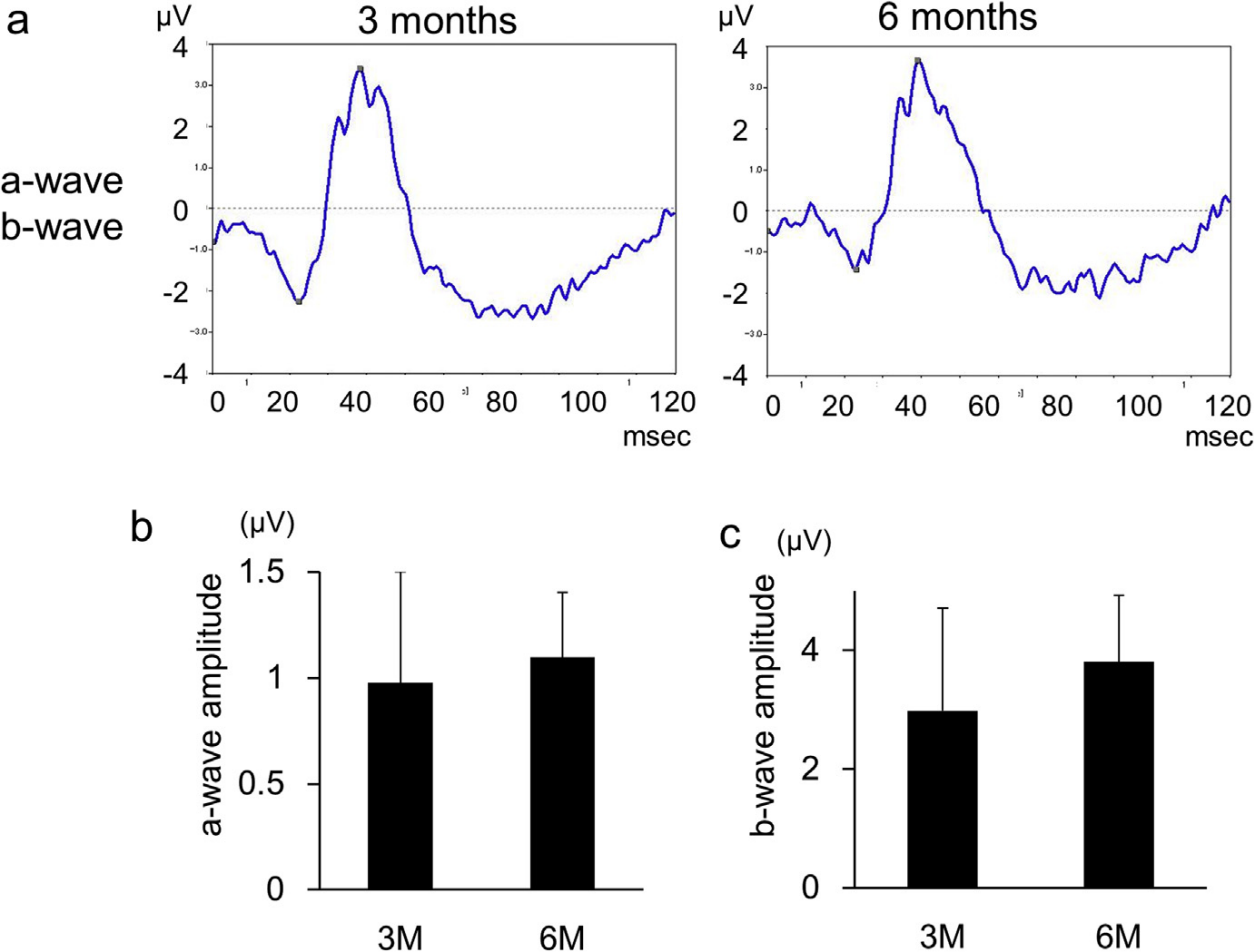
Visual functions of eyes in cynomolgus monkeys. a) a- and b-waves in the focal macular electroretinogram examination for the right eye of a 23-year-old cynomolgus monkey, performed at 3 and 6 months after the start of KUS121-administration. b, c) Graphs showing average amplitudes of a- and b-waves of the focal macular electroretinograms (b, c, respectively) at 3 and 6 months after start of KUS121 administration. The time-dependent changes are not significant in the a- and b-waves, respectively. Error bar: standard deviation.

To evaluate the potential side effects of KUS121 on the ocular and systemic status of cynomolgus monkeys, we performed focal electroretinography, blood sampling, and body weight measurements at multiple evaluation points. At 3 months after the start of administration, the amplitudes of a- and b-wave were 0.98 ± 0.53 μV and 2.98 ± 1.73 μV, respectively, and at 6 months after administration, these were 1.10 ± 0.31 μV and 3.81 ± 1.12 μV, which were not longitudinally different (*p* = 0.605 and 0.300, respectively, paired *t*-test, Fig. 8). Blood and urine tests to monitor general status did not show any significant changes in complete blood counts, inflammation markers, hepatobiliary and renal functions.

At the final end point, we also made histological sections of the major organs (brain, lung, liver, gastrointestinal tract, kidney, and genitals) in all five monkeys. None of these examinations showed any obvious abnormal findings, suggesting that there were no adverse effects of KUS121.

## 3 Discussion

We have recently developed specific inhibitors of the ATPase activities of VCP, naming them KUSs or ATP regulators [13]. KUSs have been able to effectively mitigate pathological phenotypes in the diseased retinas of several animal models by suppressing decreases in cellular ATP levels and consequently reducing ER stress and cell death [13, 14, 16, 18]. Notably, the efficacy of KUSs was observed not only in the inner retina, but also in the outer retina.

In the present study, we showed that KUSs can maintain the function of retinal cells as well as protect them from cell death. In particular, KUSs can prevent or clear drusen. In this regard, we examined the effect of KUS121 on pathology in CCR2-deficient mice, a mouse model of AMD, showing that systemic administration of KUS121 prevented the accumulation of drusen-like lesions and vacuolization in RPE cells, with significant preservation of visual function. Furthermore, we found that ER stress was raised in all retinal layers in aged CCR2-deficient mice, and that it was significantly suppressed by KUS121. We also confirmed that long-term systemic administration of KUS121 was quite safe in monkeys with drusen, which are thought to be the closest animal model to human AMD. Moreover, KUS121 tended to decrease drusen in the monkeys as well, although the effect was not significant.

AMD is a leading cause of blindness in developed countries, and there is no effective treatment, especially for the atrophic type [11]. Drusen [22] and/or subretinal drusenoid deposits, as well as ER stress around the RPE layer [5], are commonly observed as preclinical symptoms and a core pathological event, respectively, in patients with AMD. Thus, prevention or clearance of these abnormal deposits, and/or removal of ER stress, are considered the most effective therapeutic strategies to protect patients from vision loss.

To date, no valid animal models have reproduced all characteristics of AMD. However, CCL2- or CCR2-deficient mice mimic the chorioretinal pathologies of AMD [17]. CCL2 and CCR2 encode a chemokine ligand and its receptor, respectively, and it has been speculated that macrophage function is reduced in CCL2- or CCR2-deficient mice. Thus, these mice would have trouble scavenging cellular waste products, particularly those from retinal cells. We confirmed, using light microscopy, that aged CCR2-deficient mice had degenerated RPE cells with vacuoles, as reported previously [17]. Furthermore, we showed basal laminar deposition and Bruch's membrane thickening using electron microscopic examination (Figs. 1 and 3). These pathological changes were not evident in the corresponding areas of the age-matched wild-type mice. Significantly, administration of KUS121 mitigated these pathological features in the RPE or Bruch's membrane of aged CCR2-deficient mice (Fig. 3). In addition, KUS121 significantly preserved retinal function (Fig. 4). Consistent with an independent report that CCR2-deficient mice do not always exhibit the typical traits of wet AMD, such as choroidal neovascularization and retinal exudation [18], we did not observe any neovascular tissue using fundus photography and optical coherence tomography up to 15 months of age. Therefore, the efficacy of KUS121 on neovascular lesions will require further investigation.

Most surprisingly, perhaps, was that ER stress was evoked in all retinal cell layers, as revealed by immunohistochemical examination of chorioretinal sections of aged CCR2-deficient mice, yet we did not observe evidence of cell death in any type of retinal neuronal cells. These observations were quite different from those in other retinal disease models, in which ER stress is restrictively evoked in the affected retinal cells (ganglion cells in glaucoma models and photoreceptor cells in retinitis pigmentosa models), leading to cell death in the affected cells. Interestingly, KUS121 substantially suppressed age-dependent ER stress in all retinal cell layers, *i.e.* not only in the outer retina, but also in the inner retina (Fig. 5). We did not check the concentration of KUS121 in the retina or choroid. As discussed above, we have known that the efficacy of KUS121 is well correlated with suppression of ER stress. Suppression of ER stress in the retinas of KUS121-treated CCR2 deficient mice (Fig. 5) implies that orally administered KUS121 is effectively delivered to the eyes in the experiment. In other mouse models, we have repeatedly shown profound coupling of ATP decreases, ER stress, and cell death. In this model, we confirmed the efficacy of KUS121. However, cell death was not observed in the neural retina. It thus appears that in the affected retina, slight ATP decreases, which were sufficient to induce ER stress but not to trigger cell death, were the underlying cause of functional decreases in the retinal cells, especially the RPE.

In human AMD, such RPE dysfunction in the early phase of the disease would lead to degeneration in the macular photoreceptors (cones and rods), followed by irreversible loss of vision in the late phase of the disease. Importantly, this causative ATP decrease would be canceled by KUS121. The effects of KUS121, confirmed in CCR2-deficient mice, were consistent with our previous data addressing the compound's therapeutic efficacy on animal models of glaucoma and retinitis pigmentosa [13, 14, 15], indicating that KUSs may be useful in the treatment of RPE abnormalities and AMD, not only in glaucoma and retinitis pigmentosa.

Finally, we administrated KUS121 to cynomolgus monkeys with drusen. The monkey model is valuable, because the monkey has a macula, and the retina's physical structure and metabolism are close to those of humans. Due to the experimental cost, we could use only five monkeys. Thus, the effect of KUS121 on the drusen was not apparent. However, it is notable that we did not observe any presumptive ocular or systemic side effects during systemic administration of KUS121 for 9 months. Using the stored blood and urine samples for monitoring general health status, we measured concentrations of KUS121. As a result, KUS121 concentrations in the plasma ranged from 0.4–3.1 ng/mL, and those in the urine were 64–1568 ng/mL. The concentration in the plasma was generally very low. We think the reason could be that the samples were collected under general anesthesia before feeding, when the KUS121 concentration was the lowest. Readily detectable levels of KUS121 were observed in the collected urine, indicating that substantial amounts of KUS121 were delivered in the blood. We have known, from our rodent experiments, that when KUS121 is delivered in the blood, KUS121 is efficiently delivered to the eyes, including the retinas. Taken together, these observations suggest that KUS121 was efficiently delivered to the eyes in the experiments.

No promising therapy has been established for atrophic type AMD, despite numerous trials that have included targeting the complement system and stem cell therapy. Therefore, in place of neurotrophic factors, visual cycle modulators, anti-oxidants, and anti-inflammatory therapy [23], KUS121 is a promising new strategy in the treatment of drusen, and in the prevention of chorioretinal atrophy in late AMD. Nonetheless, it is still necessary to conduct a well-planned, step-wise sequence of preclinical studies.

## 4 Materials & methods

### 4.1 Experiments in CCR2-deficient mice

All studies were conducted in compliance with the ARVO Statement for the Use of Animals in Ophthalmic and Vision Research. All protocols were approved by the Institutional Review Board of Kyoto University Graduate School of Medicine (MedKyo 11229, 12245, 13221, 14213, 15522, 16213). CCR2-deficient mice [17] were obtained from the Jackson Laboratory (Bar Harbor, ME, USA), and genotyped using PCR. To enhance visualization of the RPE and choroid by immunohistochemical analysis in the retina of CCR2-deficient mice, we backcrossed CCR2-deficient mice with albino mice repeatedly until melanin pigments were lost from the eyes. The environment was maintained at a 14-hour light/10-hour dark cycle. All mice were fed *ad libitum*. Before the fundus images or electroretinograms were acquired, the mice were anesthetized by intramuscular injection of a ketamine (70 mg/kg)/xylazine (14 mg/kg) mixture. Their pupils were dilated to a diameter of approximately 2 mm using tropicamide and phenylephrine (0.5% each) eye drops.

### 4.2 Administration of KUS121 in CCR2-deficient mice

Six-month-old CCR2-deficient mice were assigned to either the KUS121-treatment group (*n* = 15) or the control group (*n* = 11). Mice in the KUS121-treatment group had *ad libitum* access to water containing 384.5 mg/L of KUS121, while those in the control group had *ad libitum* access to water without KUS121 from 9 months to 15 months of age. In the experiment assessing visual function, CCR2-deficient mice that were assigned to the KUS121-treatment group (*n* = 12) were administered KUS121 orally from 2 months to 9 months of age; they also had *ad libitum* access to water containing 384.5 mg/L of KUS121. CCR2-deficient mice assigned to the control group (*n* = 15) were administered the vehicle orally (5% Cremophor/phosphate buffered saline). Empirically, adult mice drank 0.14–0.22 mL/day/kg of water, while in reality some of the water was spilt in the cage. Thus, the actual administration was less than 80 mg/kg/day with *ad libitum* access and was estimated to be 50–70 mg/kg/day with *ad libitum* access and between 100 mg/kg/day to 120 mg/kg/day with *ad libitum* access combined with oral administration with feeding tubes.

### 4.3 Ophthalmic evaluations in CCR2-deficient mice

Color fundus photography (Micron™; Phoenix research lab, CA, USA) centered on the optic disc and focusing on the deep retina and RPE layer was performed at each evaluation point. The number of round yellowish lesions was manually counted three separate times by masked evaluators at each evaluation point; the averaged values were then analyzed.

Scotopic electroretinography was performed using 9-month-old CCR2-deficient mice. The mice were dark-adapted overnight before anesthetization. The electroretinograms were recorded using a gold loop corneal electrode with a light-emitting diode (Mayo Corp., Inazawa, Japan). A reference electrode was placed in the mouth, and a ground electrode was inserted into the anus. The electroretinography response signals were amplified, digitized at 10 kHz with a band-pass filter of 0.3–500 Hz, and analyzed using a PowerLab 2/25 (AD instruments, New South Wales, Australia). The b-wave amplitudes of the rod response (at a stimulus intensity of 0.01 cds/m^2^ [ISCEV standard [24]]; dark-adapted 0.01), and the a-wave amplitudes of the mixed cone and rod response (at a stimulus intensity of 3.0 cds/m^2^ [ISCEV standard [24]]; dark-adapted 3) were analyzed.

SD-OCT images were obtained using a custom-made, speckle noise-reduced SD-OCT scanner with eye-tracking function based on that of the Spectralis HRA+OCT (*Multiline* OCT [25, 26]; Heidelberg Engineering, Heidelberg, Germany). A 25-diopter adaptor lens was placed on the objective lens of the *Multiline* OCT to focus on the mouse retina.

### 4.4 Histological study in CCR2-deficient mice

After ophthalmic examination at the final evaluation point, the mice were euthanized using pentobarbital overdose and their eyeballs were enucleated. The eyes were then fixed in 4% paraformaldehyde for 24 hours at 4 °C and subsequently embedded in paraffin. Serial 6-μm paraffin sections were then cut. Selected retinal sections were stained with hematoxylin and eosin. We used histological sections from albino-CCR2-deficient mice for immunostaining with an anti-CHOP antibody. They were then photographed under a microscope (BZ-9000; Keyence, Osaka, Japan).

### 4.5 Electron microscopy in CCR2-deficient mice

After fixation, paraffin sections were fixed in 1% osmium tetroxide for 90 minutes. The retinas were dehydrated through a graded series of ethanol (50%–100%), cleared in propylene oxide, and embedded in epoxy resin. Ultrathin sections were cut using an ultramicrotome and stained using uranyl acetate and lead citrate. The stained sections were then observed under transmission electron microscopy (H-7650; Hitachi Co., Tokyo, Japan).

### 4.6 Western blotting

To obtain chorioretinal samples from mice for western blotting, eyeballs from 15-month-old CCR2-deficient or wild-type mice were enucleated after pentobarbital overdose. A mixture of the RPE, choroid, and sclera (RPE/choroid) were collected as described previously [14]. Eyes from 15-month-old CCR2-deficient mice in the KUS121-treatment group and control group were analyzed. The relative intensities of bands were quantified using Image Lab 4.1 (Bio-Rad, CA, USA).

### 4.7 Antibodies

The anti-CHOP antibody was purchased from Santa Cruz Biotechnology (CA, USA), while anti-actin was sourced from Chemicon (MA, USA).

### 4.8 Experiments with cynomolgus monkeys

Two young (7 and 8 year-old) and three senile (23, 23, and 26 year-old) cynomolgus monkeys with drusen in the macular area were used in this experiment [21]. The monkeys were bred at the Tsukuba Primate Research Center (TPRC), National Institutes of Biomedical Innovation, Health and Nutrition (NIBIOHN). All the procedures were conducted according to the rules for animal care and management of the TPRC [27], the Guiding Principles for Animal Experiments Using Nonhuman Primates formulated by the Primate Society of Japan [28], and the Institute for Laboratory Animal Research (ILAR) Guide for Care and Use of Laboratory Animals [29]. The research protocol was approved by the Animal Welfare and Animal Care Committee of NIBIOHN. All animals were housed under the following conditions: temperature, 23–27 °C; humidity, 50–70%; 12 air changes/hr; 12/12-hr light/dark cycle, and fed 70 g of commercial food (CMK-2; CLEA Japan, Inc., Tokyo, Japan) and 100 g of apples daily. Tap water was supplied *ad libitum*.

All five monkeys were administered KUS121 orally at a dosage of 10 mg/kg/day (twice a day) from the start.

About 20 minutes before the ophthalmic examinations, one drop of tropicamide-phenylephrine hydrochloride was instilled into both eyes of each animal to dilate the pupils. The monkeys were then anesthetized by intramuscular injection of 10 mg/kg ketamine-HCL (Ketaral-50; Sankyo, Tokyo). In each monkey, 45° digital fundus photographs were obtained using a digital fundus camera (TRC-50LX; Topcon, Tokyo, Japan; 3,216 × 2,136 pixels) at baseline, and at 3, 6, and 9 months after the start of administration. For morphologic and functional evaluations of the macular area, SD-OCT (Spectralis HRA+OCT) and focal electroretinography (ER-80, Kowa, Tokyo, Japan) were also performed 3 and 6 months after the start of administration. In focal electroretinography recording, a Burian-Allen bipolar contact lens electrode (Hansen Ophthalmic Laboratories, Iowa City, IA) was placed in the conjunctival sac after the pupils were maximally dilated. A chloride silver electrode was attached to the left earlobe as a ground electrode. The focal electroretinography was elicited by 15° circular stimuli positioned on the fovea. It was composed of an infrared camera (Kowa) and a stimulation system (Mayo Co., Nagoya, Japan). The luminances of white stimulus light and background illumination were 181.5 and 6.9 cd/m^2^, respectively. A background field of 45° visual angle was projected to the eye from the fundus camera. The focal electroretinography was recorded with 2-Hz rectangular stimuli (150 msec with the light on and 350 msec with the light off). The 15° circular stimulus was carefully and constantly centered on the fovea, as observed through the infrared camera. The recording (100–150 responses) was made twice to confirm reproducibility, and a total of 200–300 responses were averaged by the signal processor (Neuropack MEB-2204; Nihon Kohden, Tokyo, Japan). The focal electroretinography response was digitized at 10 kHz with a band-pass filter of 1–300 Hz.

### 4.9 Statistical analysis

All values are presented as means ± standard deviations. Statistical analysis was performed using PASW Statistics version 18.0 (SPSS Inc., Chicago, IL). Variables between the KUS121-treatment and control groups were compared using the unpaired *t*-test. Analysis of variance was used to evaluate variables among multiple groups, and differences in each parameter among all examination time points. *Post-hoc* analysis was performed using paired and unpaired *t*-tests, and *p*-values were corrected using Tukey's method. The level of statistical significance was set at *p* < 0.05.

## Declarations

### Author contribution statement

Yuki Muraoka, Yuto Iida: Conceived and designed the experiments; Performed the experiments; Analyzed and interpreted the data; Wrote the paper.

Hanako Ohashi Ikeda: Conceived and designed the experiments; Analyzed and interpreted the data; Contributed reagents, materials, analysis tools or data; Wrote the paper.

Sachiko Iwai, Masayuki Hata: Conceived and designed the experiments; Performed the experiments; Analyzed and interpreted the data.

Takeshi Iwata: Conceived and designed the experiments; Contributed reagents, materials, analysis tools or data.

Mao Nakayama: Performed the experiments; Analyzed and interpreted the data.

Nobuhiro Shimozawa, Yuko Katakai: Performed the experiments.

Akira Kakizuka: Conceived and designed the experiments; Analyzed and interpreted the data; Contributed reagents, materials, analysis tools or data; Wrote the paper.

Nagahisa Yoshimura, Akitaka Tsujikawa: Conceived and designed the experiments; Contributed reagents, materials, analysis tools or data.

### Funding statement

This work was supported in part by Research grants from the Astellas Foundation for Research on Metabolic Disorders, the Japan Foundation for Applied Enzymology, the Uehara Memorial Foundation, Mochida Memorial Foundation for Medical and Pharmaceutical Research, YOKOYAMA Foundation for Clinical Pharmacology (YRY1308), Japan Intractable Diseases Research Foundation, Japan Research Foundation for Clinical Pharmacology, KOBAYASH MAGOBE MEMORIAL Medical Foundation, Takeda Science Foundation, Japan National Society for the Prevention of Blindness, Novartis Pharma, a Grant-in-Aid for Young Scientists (24791850, H.O.I), grants from SORST of JST (A.K.), Japan society for the promotion of science (JSPS KAKENHI; JP16K11285), Japan Agency for Medical Research and Development (AMED), the Ministry of Education, Culture, Sports, Science, and Technology of Japan (A.K., H.O.I. and N.Y.), the Ministry of Health, Labor, and Welfare of Japan (A.K., H.O.I., N.Y., and A.T.) and the Innovative Techno-Hub for Integrated Medical Bio-Imaging of the Project for Developing Innovation Systems (N.Y.) from the Ministry of Education, Culture, Sports, Science, and Technology of Japan.

### Competing interest statement

The authors declare the following conflict of interests: Kyoto University applied for patents (PCT/JP2014/053898 and WO2014129495 A1); Yuki Muraoka, Hanako Ohashi Ikeda, Nagahisa Yoshimura, and Akira Kakizuka were the inventors in these patents. The other authors declare no competing interests.

### Additional information

No additional information is available for this paper.
