# Supplementary figures and images for: KUS121, an ATP regulator, mitigates chorioretinal pathologies in animal models of age-related macular degeneration
Source: Heliyon. 2018 May 14;4(5):e00624. doi: 10.1016/j.heliyon.2018.e00624 (PMC5986307; doi:10.1016/j.heliyon.2018.e00624)

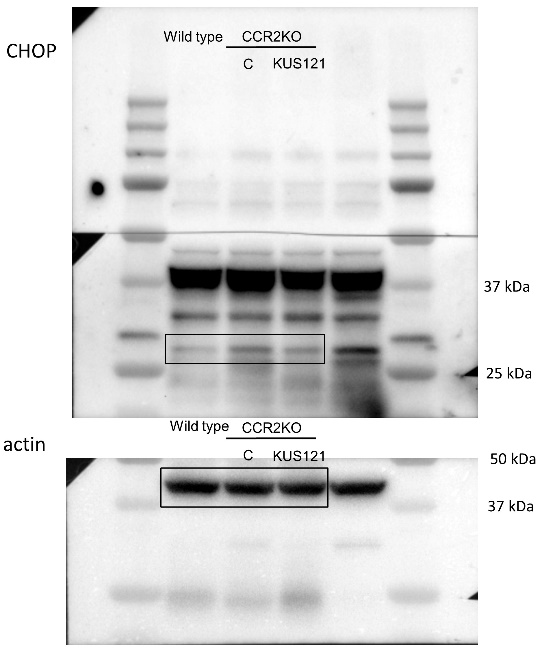


**Supplementary Figure S1.** Complete scans of the western blots presented in Fig. 4a.

Supplement: Supplementary Figure 1 [file mmc1.docx]
